# Systemic confounders affecting serum measurements of omega-3 and -6 polyunsaturated fatty acids in patients with retinal disease

**DOI:** 10.1186/s12886-016-0335-9

**Published:** 2016-09-05

**Authors:** Anima D. Bühler, Felicitas Bucher, Michael Augustynik, Jan Wöhrl, Gottfried Martin, Günther Schlunck, Hansjürgen Agostini, Daniel Böhringer, Gerhard Pütz, Andreas Stahl

**Affiliations:** 1 Eye Center, Medical Center, Faculty of Medicine, University of Freiburg, Killianstrasse 5, 79106 Freiburg, Germany; 2 Institute of Clinical Chemistry and Laboratory Medicine, University Freiburg Medical Center, Freiburg, Germany

**Keywords:** AMD, DME, RVO, Lipid, Omega-3, PUFA

## Abstract

**Background:**

Omega-3 polyunsaturated fatty acids (PUFAs) have a highly anti-angiogenic effect in animal models. However, the clinical relevance of omega-3PUFAs in human retinal pathologies remains unclear. The ARED 2 study found no effect of omega-3 PUFA supplementation on progression of age related macular degeneration (AMD). The aim of this study was to compare serum levels of omega-3- and omega-6 PUFAs between patients with diabetic retinopathy (DR), AMD and retinal vein occlusion (RVO), and to identify potential confounders of serum level measurements.

**Methods:**

Venous blood samples were collected from 44 patients with DR, 25 with AMD, 12 with RVO and 27 controls. The lipid phase was extracted and analyzed using mass spectrometry. Retinal disease staging was done by indirect funduscopy and FAG where appropriate. Patient demographics and medical history including current medication and fasting state were acquired. Tukey contrasts for multiple comparisons of the mean and linear regression analysis were used for statistical analysis.

**Results:**

Our data revealed no significant differences in omega-6 PUFA serum levels between patients with AMD, DR, RVO and controls (*p* > 0.858). Uncorrected omega-3 PUFA levels were significantly higher in patients with AMD compared to DR but not compared to controls (*p* = 0.004). However, after correcting for possible confounders such as body mass index (BMI), age, sex, fasting and use of statins, no statistically significant difference remained for serum omega-3 PUFA levels. Fasting was identified as an independent confounder of total omega-6 PUFAs, three individual omega-6 PUFAs and one omega-3 PUFA(*p* < 0.0427). Statin use was identified as an independent confounder of α-linolenic acid (an omega-3PUFA; *p* = 0.0210).

**Conclusion:**

In this pilot study with relatively low patient numbers, we report significant differences in serum levels of omega-3PUFAs among patients with different types of retinal diseases. However, these differences were not robust for disease specificity after correction for possible confounders in our cohort. Our results demonstrate that serum lipid profiles need to be interpreted with caution since they are significantly altered by variables like fasting and medication use independent from the underlying disease. Correcting for respective confounders is thus necessary to compare serum lipid profiles in clinical studies.

**Electronic supplementary material:**

The online version of this article (doi:10.1186/s12886-016-0335-9) contains supplementary material, which is available to authorized users.

## Background

Angioproliferative retinal diseases such as diabetic retinopathy or the wet type of age related macular degeneration are responsible for visual impairment among many working age individuals and the elderly [1, 2] and lipid mediators have been discussed as part of the pathogenetic cascade in these disorders [3, 4].

Omega-3-polyunsatureted fatty acids (PUFAs) are major structural lipids of retinal photoreceptor outer segment membranes and have important structural and protective functions in the retina [5]. The anti-inflammatory effects of some of these omega-3PUFAs such as eicosapentaenoic acid (EPA) and docosahexaenoic acid (DHA) were implicated to play a protective role in AMD development [5, 6]. In addition, it was also previously described that omega-3PUFAs increase the retinal density of macular pigment, which filters blue light, and has local antioxidant and anti-inflammatory activities [7].

In the mouse model of oxygen-induced retinopathy (OIR), omega-3PUFAs were found to have potent anti-angiogenic effects on retinal neovascularization [8]. For example, Sapieha et al. showed that the omega-3 PUFA metabolite 4-hydroxy-docosahexaenoic acid (4-HDHA) directly inhibits endothelial cell proliferation and sprouting angiogenesis via a 5-lipoxygenase dependent pathway. It was also shown that this effect was independent of the anti-inflammatory properties of 4-HDHA [8]. In contrast, the results of the age-related eye disease study 2 (AREDS2) [9] indicate that the addition of the omega-3PUFAs DHA and eicosapentaenoic acid (EPA) to the standard AREDS formulation does not reduce the risk of AMD progression.

Different from AREDS2, however, Merle et al.found serum EPA to be associated with a lower risk for neovascular AMD in 290 patients from the Nutritional AMD Treatment 2 Study (NAT2) who had neovascular AMD in one eye and early AMD in the other eye [10]. Merle et al. also observed in a different study with 936 elderly patients that high plasma omega-3 PUFA levels were associated with a reduced risk for late AMD [11]. In contrast, Kabasawa et al. detected no difference in fatty acid profiles of AMD patients, except for a mild association of EPA with all types of AMD investigated [12].

These contradicting results of various AMD studies demonstrate the need for characterization of confounding parameters which might affect lipid measurements in serum samples. This is not only important for AMD research but also for research in other areas of retinal disease where lipid imbalance may play a role such as diabetic retinopathy (DR) and retinal vein occlusion (RVO). To date, significantly less scientific data exist for DR and RVO compared to AMD with regard to the potential effect of lipids on disease development or progression.

The aim of our study was therefore to identify potential confounders which may affect a perceived correlation of omega3 PUFAs with the degree of AMD, DR or RVO. In addition to omega-3 PUFAs, we also investigated omega-6 PUFAs as well as monounsaturated and saturated fatty acids.

## Methods

Venous blood samples were obtained from 81 patients with DR, AMD or RVO. In addition, samples from 27 control patients without any of the above diseases were collected. Patients who had more than one of the mentioned pathologies were excluded. Diabetic patients were sub-classified in five groups: diabetes without DR (5 patients), mild non-proliferative DR (6 patients), moderate non-proliferative DR (14 patients), severe non-proliferative DR (5 patients) and proliferative DR (15 patients). The grading was done according to the EDTRS–guidelines [13]. Patients with AMD were sub-grouped in four different categories: early dry AMD (4 patients), active wet AMD (12 patients), scarred wet AMD (3 patients) and geographic atrophy (GA; 6 patients). Patients with retinal vein occlusion were distinguished as suffering from branch retinal vein occlusion (BRVO, 7 patients), or central retinal vein occlusion (CRVO, 5 patients).

Additional clinical data were acquired by indirect fundoscopy and FAG where appropriate. For all patients additional information was gathered including medical history, current and past medication (including vitamin supplementation), state of fasting and body mass index (BMI).

The study was registered with the Ethics Committee at the University Hospital Freiburg. After obtaining written informed consent, one serum sample per patient was acquired by venous blood draw and stored at −80 °C until being used for lipid extraction. Lipid extraction was performed by the Department of Clinical Chemistry at the University of Freiburg as previously described at a temperature of 4 ° C in a two-step procedure [14]. 100 μl of serum sample were mixed with 100 μl methylpentadecanoate 5 mg/l as internal lipid standard mixture (analytical standard grade, Sigma, Taufkirchen, Germany). Samples were extracted with 990 μl chloroform/methanol (17:1, V/V) for 120 min. The lower organic 17:1 lipid phase extract was collected. The remaining aqueous sample material was re-extracted with 990 μl chloroform/methanol (2:1, V/V) for 120 min. Again, the lower organic 2:1 lipid phase extract was collected. Combined organic phases were then evaporated in vacuum. The dried lipid extracts were dissolved in 70 μl chloroform and 30 μl of trimethylsulfonium hydroxid (0.25 M in methanol, Sigma Aldrich) were added. To yield respective fatty acid methyl esters, the mixture was stirred for 30 min at room temperature [15]. Fatty acid methyl esters were analyzed by GC-MS on an Agilent GC 6890/MS 5973 platform (Agilent Technologies Deutschland GmbH, Böblingen Germany). Helium (5.0) was used as carrier gas, injection volume was 1 μl and flowrate was 0.4 ml/min on a SP-2380 capillary column (Sigma). Initial temperature was 100 °C, rising with 6 °C/min to a final temperature of 220 °C, which was kept for 10 min. Mass spectra were recorded in fullscan mode from m/z 45 to 800. Total ion chromatograms (TICs) and fragmentation patterns were acquired by G1701DA GC/MSD Chemstation software. Fatty acid methyl esters were identified by retention time and characteristic mass-to-charge ratios compared to a standard mass chromatogram in the NIST (National Institute of Standards and Technology) mass spectral library. Peak areas were quantitated manually for each identified fatty acid methyl ester. Results for respective fatty acids are presented as percentage of total fatty acid content per sample. Statistical analysis was performed using R with the following packages: plyr, reshape, car, Hmisc, ggplot2 and multcomp. We applied ANOVA, Tukey contrasts for between-group comparisons and linear regression for inference analyses.

## Results

Our initial analysis of native sample readings without correction for potential confounders suggested significantly higher omega-3PUFA levels in patients with AMD compared to patients with DR (*p* = 0.00405; Table 1a). For omega-6 PUFA levels, there was no significant difference between patients with AMD, DR, RVO and controls (*p* > 0,858; Table 1b).

**Table 1:** Serum omega-3 (A) and omega-6 (B) PUFA levels in patients with different retinal diseases

	Estimate	std. error	t value	Pr (>|t|)
A. omega-3 PUFAs				
AMD vs Control	0.48	0.25	1.92	0.222
DR vs Control	−0.28	0.22	−1.26	0.585
RVO vs Control	−0.13	0.31	−0.41	0.976
DR vs AMD	−0.76	0.22	−3.47	0.004**
RVO vs AMD	−0.61	0.31	−1.98	0.199
RVO vs DR	0.15	0.29	0.53	0.950
B. omega-6 PUFAs				
AMD vs Control	0.59	1.60	0.37	0.983
DR vs Control	1.11	1.42	0.79	0.858
RVO vs Control	0.64	2.02	0.32	0.988
DR vs AMD	0.52	1.44	0.37	0.983
RVO vs AMD	0.06	2.03	0.03	1.000
RVO vs DR	−0.47	1.90	−0.25	0.994

The table displays group comparisons without correction for potential confounders. p-values are shown in the right column and were calculated using ANOVA with Tukey contrasts correcting for multiple testing

**signifies *p*-values < 0.01

**Fig. 1:**
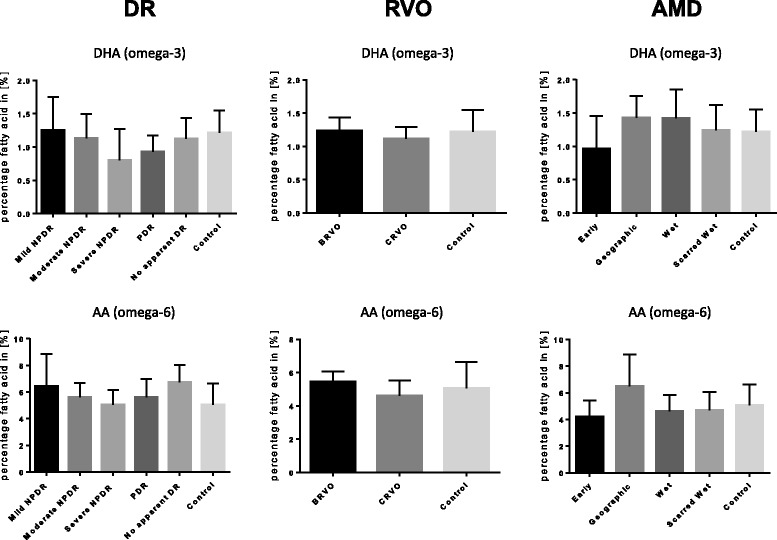
Serum patterns for two exemplary PUFAs for patients with retinal disease and controls. Top row shows serum levels of docosahexaenoic acid (DHA, 22:6), an omega-3 fatty acid, bottom row shows the same for arachidonic acid (AA, 20:4), an omega-6 fatty acid. In this analysis, patients with diabetic retinopathy (DR), retinal vein occlusion (RVO) and age-related macular degeneration (AMD) were subdivided into their respective subgroups as indicated. No significant differences were detected across groups. The same is true for all other PUFAs investigated (not shown). Mean and standard deviations of relative fatty acid compositions (all lipid classes) are given

When analyzing not only the broad disease categories AMD, DR and RVO, but also disease subgroups, there were no significant differences for omega-3 or omega-6 PUFAs. Two exemplary comparisons across disease subgroups are shown in Fig. 1 for DHA, a major omega-3 fatty acid and arachidonic acid (AA), a major omega-6 fatty acid. Similar patterns without apparent differences across disease subgroups were observed for other individual fatty acids.

Since the primary objective of this study was to determine whether independent variables may act as confounders of PUFA serum measurements, we analyzed our data set using a linear regression model correcting for patient age and sex, body mass index (BMI), the use of oral statin medication and the state of fasting (defined as at least six hours fasting prior to blood sampling). The results shown in Table 2 demonstrate that fasting state is an independent confounder for omega-6, but not omega-3 PUFA measurements. More importantly, the data also demonstrate that none of the three disease entities (DR, RVO, AMD) acts as an independent variable on total omega-3 or omega-6 PUFA levels in our cohort when using a linear regression model. A full list of fatty acids analyzed can be found as Additional file 1.

**Table 2:** Serum omega-3 (A) and omega-6 (B) PUFA levels corrected for potential confounders

	Estimate	std. error	*t* value	Pr (>|t|)
A. omega-3 PUFAs				
BMI	0.01	0.02	0.38	0.706
Statin use	0.03	0.25	0.12	0.905
Fasting	−0.37	0.29	−1.29	0.201
Age	0.01	0.01	0.55	0.582
Sex	−0.10	0.21	−0.48	0.635
AMD	0.45	0.31	1.43	0.155
DR	−0.35	0.27	−1.29	0.199
RVO	−0.23	0.35	−0.65	0.516
B. omega-6 PUFAs				
BMI	−0.19	0.12	−1.55	0.125
Statin use	−2.16	1.39	−1.56	0.123
Fasting	−5.67	1.63	−3.49	0.001***
Age	0.08	0.04	1.90	0.061
Sex	0.79	1.15	0.68	0.496
AMD	−1.82	1.75	−1.04	0.300
DR	0.37	1.45	0.26	0.798
RVO	−2.22	1.97	−1.19	0.262

When serum lipid measurements were corrected for the possible confounders body mass index (BMI), statin use, fasting, age and sex of the patient, none of the retinal disease categories acted as an independent variable affecting serum lipid levels. Fasting, in contrast, was identified as an independent variable that significantly affects serum omega-6 PUFA measurements.

***signifies *p*-values ≤ 0.001

Next, we investigated whether individual fatty acids were significantly affected by individual patient variables. Table 3 demonstrates that the fasting state of a patient acts as an independent confounder of total omega-6PUFAs, three individual omega-6 PUFAs and one omega-3 PUFA (*p* < 0.0427). Statin medication was identified as an independent confounder of α-linolenic acid (an omega-3 PUFA; *p* = 0.0210). Interestingly, patient age and BMI were no independent confounders of serum lipid measurements. The small patient number especially in the RVO subgroup has to be considered when interpreting the results of this pilot study.

**Table 3:** Independent patient variables affecting fatty acid measurements

Variable	Fatty acid affected by independent variable
BMI	–
Statin use	18:3 (Ώ-3-PUFA)
Fasting	14:00, 18:2 (Ώ-6-PUFA), α18:3 (Ώ-3-PUFA), 20:2 (Ώ-6-PUFA), 20:4 (Ώ-6-PUFA), total Ώ-6-PUFAs
Age	–
Sex	16:00

Multiple regression analysis for individual fatty acids identified several fatty acids whose serum levels were significantly associated with differences in a patient’s baseline variables. Fasting state was the most signficiant variable, independently affecting various saturated and unsaturated fatty acid measurements. Statin use and patient sex each affected one of the fatty acids investigated. Patient age and body mass index (BMI) were no independent variables of serum fatty acid measurements in our cohort. Affected fatty acids in this table are noted according to the carboxyl reference system

## Discussion

Numerous clinical studies have investigated a possible protective effect of omega-3PUFA supplementation on the progression of AMD. These studies yielded variable and contradicting results [9, 10, 16, 17]. The AREDS2 clinical trial, for example, did not find a beneficial effect for omega-3 PUFA supplementation on disease progression [9]. Other studies, in contrast, observed beneficial associations of certain omega-3 PUFA subtypes, such as EPA with AMD risk [10]. For DR and RVO there is significantly less literature compared to AMD regarding a potential association of omega-3 PUFAs with disease stage or progression. In this study, we describe important confounders that may contribute to contradicting results in studies correlating serum lipid levels and eye disease. In addition, our results add valuable data on the less studied disease entities DR and RVO with regard to systemic lipid profiles.

Despite the small sample size of this pilot study, our data clearly suggest that serum lipid levels cannot be used for clinical ophthalmological studies without vigorous correction for potential confounders. The most important confounder is patient fasting state, which affects various unsaturated and saturated fatty acid levels. Most of the existing AMD studies have corrected for potential confounders and report fasting status in their publications. For example, the study by Merle et al. which identified EPA as a risk variable for neovascular AMD did correct for a list of potential confounders including fasting state of the patient [10]. It has to be kept in mind when interpreting lipid studies in general, that not only fasting state but dietary habits in general differ between populations and can potentially impact study results. A meta-analysis published in 2008, for example, postulates reduced risk for AMD in patients with high dietary intake of Omega-3 PUFAs [18]. For a Japanese AMD population, in contrast, Kabasawa et al. reported no associations with serum omega-3 and omega-6 PUFA levels [12]. Since fish intake in Japan is generally among the highest in the world [19, 20], the effect of omega-3 PUFAs on AMD could potentially have been masked in this population. In addition, changes in serum fatty acid profiles do not necessarily translate into changes in retinal fatty acid profiles.

The second important finding of our study is that, in our cohort, total omega-3 or omega-6 PUFA levels were not independently associated with the presence of AMD. Different from our study, Merle et al. found associations between AMD and EPA, an omega-3 PUFA [10]. While Merle et al. only investigated patients with neovascular AMD, our study comprised samples from patients with all forms and stages of AMD. The subset of neovascular AMD patients might thus have been too small to pick up potential differences in AMD levels. In contrast to AMD, there is significantly less data available correlating omega-3 and omega-6 PUFA serum levels with the degree of DR or RVO. With regard to RVO, this study is to our knowledge the first to investigate possible associations with serum lipid profiles. In our relatively small patient cohort, we did not find associations of omega-3 or −6 PUFA levels and the presence of RVO. Similarly, DR was not associated with omega-3 or −6 PUFA levels in our cohort. This is different from saturated fatty acids, for which a positive association with the presence and severity of DR was described [21].

We would like to point out that larger patient numbers would be necessary in order to more reliably investigate clinically relevant differences between patient groups. The main purpose of this manuscript, however, was to identify and flag potent confounders of lipid serum measurement that have to be accounted for when performing these studies. Even with our low patient numbers we were able to identify such confounders – an indication of their potency in affecting results from serum lipid measurements. In this respect, our current manuscript has to be viewed as preparatory work for larger studies by providing important information on methodological aspects of serum lipid measurements that have to be accounted for in larger clinical trials.

## Conclusions

In summary, our data together with the discussed literature emphasize the need for (i) correction for identified confounders of serum lipid profile measurements and (ii) interpretation of lipid profiles in light not only of the disease entity studied but also the underlying patient population. One limitation of our study is the relatively low sample number. Larger patient numbers are thus needed to obtain a full understanding of all factors influencing serum lipid measurements.

## Additional file

Additional file 1: This file contains a list of all fatty acids analyzed for this study. (DOC 41 kb)

## Abbreviations

4-HDHA: 4-hydroxy-docosahexaenoic acid
AA: Arachidonic acid
AMD: Age-related macular degeneration
AREDS: Age-related eye disease study
BMI: Body mass index
BRVO: Branch retinal vein occlusion
CRVO: Central retinal vein occlusion
DHA: Docosahexaenoic acid
DR: Diabetic retinopathy
EPA: Eicosapentaenoic acid
NAT2: Nutritional AMD Treatment Study
OIR: Oxygen-induced retinopathy
PUFA: Polyunsaturated fatty acid
RVO: Retinal vein occlusion
