# Supplementary material for: Systemic confounders affecting serum measurements of omega-3 and -6 polyunsaturated fatty acids in patients with retinal disease
Source: BMC Ophthalmol. 2016 Sep 5;16(1):159. doi: 10.1186/s12886-016-0335-9 (PMC5011975; doi:10.1186/s12886-016-0335-9)
Supplement: Additional file 1: — This file contains a list of all fatty acids analyzed for this study. (DOC 41 kb) [file 12886_2016_335_MOESM1_ESM.doc]

**Fatty acid: 12:00**

Estimate Std. Error t value Pr(>|t|)

(Intercept) 0.1963684 0.1494700 1.314 0.1924

BMI 0.0050368 0.0044942 1.121 0.2655

statin use 0.0194862 0.0504994 0.386 0.7005

fasting -0.1145663 0.0603468 -1.898 0.0610 .

age -0.0001726 0.0016659 -0.104 0.9177

AMD 0.1068398 0.0658852 1.622 0.1085

DR -0.1060552 0.0530893 -1.998 0.0489 *

RVO 0.0271140 0.0707949 0.383 0.7027

sex 0.0004727 0.0420776 0.011 0.9911

---

Signif. codes: 0 ‘***’ 0.001 ‘**’ 0.01 ‘*’ 0.05 ‘.’ 0.1 ‘ ’ 1

**Fatty acid: 14:00**

Coefficients:

Estimate Std. Error t value Pr(>|t|)

(Intercept) 1.0536748 0.6065598 1.737 0.0859 .

BMI 0.0342611 0.0182194 1.880 0.0634 .

statin use 0.0288914 0.2045069 0.141 0.8880

fasting -0.4883129 0.2449422 -1.994 0.0493 *

age 0.0005337 0.0067330 0.079 0.9370

AMD 0.1766874 0.2618437 0.675 0.5016

DR -0.4705011 0.2143724 -2.195 0.0308 *

RVO 0.1142246 0.2872145 0.398 0.6918

sex 0.2611466 0.1687784 1.547 0.1254

---

Signif. codes: 0 ‘***’ 0.001 ‘**’ 0.01 ‘*’ 0.05 ‘.’ 0.1 ‘ ’ 1

**Fatty acid: 16:00**

Coefficients:

Estimate Std. Error t value Pr(>|t|)

(Intercept) 28.10424 4.96671 5.659 1.88e-07 ***

BMI 0.11368 0.14919 0.762 0.4481

statin use -3.07858 1.67457 -1.838 0.0694 .

fasting 1.54871 2.00567 0.772 0.4421

age -0.08614 0.05513 -1.562 0.1218

AMD 4.66554 2.14406 2.176 0.0322 *

DR 4.58513 1.75535 2.612 0.0106 *

RVO 3.05434 2.35181 1.299 0.1974

sex 2.91456 1.38201 2.109 0.0378 *

---

Signif. codes: 0 ‘***’ 0.001 ‘**’ 0.01 ‘*’ 0.05 ‘.’ 0.1 ‘ ’ 1

**Fatty acid: 17:00**

Coefficients:

Estimate Std. Error t value Pr(>|t|)

(Intercept) 2.86891 1.46347 1.960 0.0531 .

BMI -0.02817 0.04396 -0.641 0.5233

statin use 0.03696 0.49342 0.075 0.9405

fasting 0.39810 0.59098 0.674 0.5023

age -0.01209 0.01624 -0.744 0.4587

AMD -0.52913 0.63176 -0.838 0.4046

DR -0.59620 0.51722 -1.153 0.2522

RVO -0.72074 0.69297 -1.040 0.3012

sex -0.06823 0.40722 -0.168 0.8673

---

Signif. codes: 0 ‘***’ 0.001 ‘**’ 0.01 ‘*’ 0.05 ‘.’ 0.1 ‘ ’ 1

**Fatty acid: 18:00**

Coefficients:

Estimate Std. Error t value Pr(>|t|)

(Intercept) 15.660486 4.811744 3.255 0.00161 **

BMI -0.071563 0.144532 -0.495 0.62174

statin use 0.707311 1.622321 0.436 0.66391

fasting 3.315790 1.943088 1.706 0.09145 .

age 0.053387 0.053412 1.000 0.32028

AMD -4.223861 2.077165 -2.033 0.04502 *

DR -0.004094 1.700583 -0.002 0.99808

RVO -3.262190 2.278428 -1.432 0.15575

sex -1.339648 1.338893 -1.001 0.31978

---

Signif. codes: 0 ‘***’ 0.001 ‘**’ 0.01 ‘*’ 0.05 ‘.’ 0.1 ‘ ’ 1

**Fatty acid: 16:1 (n-7)**

Coefficients:

Estimate Std. Error t value Pr(>|t|)

(Intercept) -4.41568 7.91202 -0.558 0.5801

BMI 0.07788 0.22245 0.350 0.7283

statin use 3.84022 2.30003 1.670 0.1034

fasting 6.58524 3.85236 1.709 0.0958 .

age 0.10554 0.08169 1.292 0.2044

AMD -5.72549 2.88421 -1.985 0.0546 .

DR -4.82820 2.49999 -1.931 0.0611 .

RVO -5.43442 3.26647 -1.664 0.1046

sex 0.55592 2.03342 0.273 0.7861

---

Signif. codes: 0 ‘***’ 0.001 ‘**’ 0.01 ‘*’ 0.05 ‘.’ 0.1 ‘ ’ 1

**Fatty acid: 16:1 (n-9)**

Coefficients:

Estimate Std. Error t value Pr(>|t|)

(Intercept) -2.25343 5.36328 -0.420 0.6765

BMI 0.11216 0.16047 0.699 0.4884

statin use 3.99950 2.03624 1.964 0.0562 .

fasting -0.07326 2.65523 -0.028 0.9781

age 0.01987 0.05645 0.352 0.7266

AMD 0.54036 2.22344 0.243 0.8092

DR -0.60359 1.64751 -0.366 0.7159

RVO 6.27312 2.57529 2.436 0.0192 *

sex -1.18916 1.47475 -0.806 0.4246

---

Signif. codes: 0 ‘***’ 0.001 ‘**’ 0.01 ‘*’ 0.05 ‘.’ 0.1 ‘ ’ 1

**Fatty acid: 18:1**

Coefficients:

Estimate Std. Error t value Pr(>|t|)

(Intercept) 23.60555 4.38142 5.388 6.01e-07 ***

BMI 0.06491 0.13142 0.494 0.6226

statin use -0.40758 1.47703 -0.276 0.7832

fasting 0.47263 1.76774 0.267 0.7898

age -0.05959 0.04905 -1.215 0.2277

AMD 3.39572 1.89144 1.795 0.0761 .

DR 0.79377 1.55345 0.511 0.6107

RVO 1.90370 2.07169 0.919 0.3607

sex 1.27761 1.22553 1.042 0.3001

---

Signif. codes: 0 ‘***’ 0.001 ‘**’ 0.01 ‘*’ 0.05 ‘.’ 0.1 ‘ ’ 1

**Fatty acid: 20:1**

Coefficients:

Estimate Std. Error t value Pr(>|t|)

(Intercept) 0.1893781 0.0683743 2.770 0.00687 **

BMI 0.0007083 0.0020551 0.345 0.73121

statin use -0.0007281 0.0236388 -0.031 0.97550

fasting -0.0396670 0.0276013 -1.437 0.15431

age 0.0002410 0.0007598 0.317 0.75190

AMD -0.0208407 0.0294987 -0.706 0.48179

DR -0.0033023 0.0242849 -0.136 0.89215

RVO 0.0281542 0.0332451 0.847 0.39942

sex -0.0079659 0.0193669 -0.411 0.68186

---

Signif. codes: 0 ‘***’ 0.001 ‘**’ 0.01 ‘*’ 0.05 ‘.’ 0.1 ‘ ’ 1

**Fatty acid: 18:2**

Coefficients:

Estimate Std. Error t value Pr(>|t|)

(Intercept) 15.92563 3.78738 4.205 6.26e-05 ***

BMI -0.19912 0.11376 -1.750 0.08355 .

statin use -1.44037 1.27695 -1.128 0.26240

fasting -4.48126 1.52943 -2.930 0.00432 **

age 0.07964 0.04204 1.894 0.06145 .

AMD -1.19166 1.63496 -0.729 0.46802

DR 0.11386 1.33855 0.085 0.93240

RVO -0.30244 1.79338 -0.169 0.86647

sex 0.22328 1.05386 0.212 0.83270

---

Signif. codes: 0 ‘***’ 0.001 ‘**’ 0.01 ‘*’ 0.05 ‘.’ 0.1 ‘ ’ 1

**Fatty acid: 18:2 (omega)**

Coefficients:

Estimate Std. Error t value Pr(>|t|)

(Intercept) 0.081204 0.122144 0.665 0.508

BMI 0.005581 0.003671 1.520 0.132

statin use -0.032988 0.042181 -0.782 0.436

fasting -0.024533 0.049300 -0.498 0.620

age 0.001072 0.001355 0.791 0.431

AMD 0.080601 0.052702 1.529 0.130

DR -0.007474 0.043147 -0.173 0.863

RVO 0.012216 0.059392 0.206 0.838

sex 0.029278 0.034476 0.849 0.398

---

Signif. codes: 0 ‘***’ 0.001 ‘**’ 0.01 ‘*’ 0.05 ‘.’ 0.1 ‘ ’ 1

**Fatty acid: 20:2**

Coefficients:

Estimate Std. Error t value Pr(>|t|)

(Intercept) 0.091852 0.150911 0.609 0.54434

BMI 0.002129 0.004536 0.469 0.64005

statin use -0.015677 0.052116 -0.301 0.76427

fasting -0.177411 0.060911 -2.913 0.00455 **

age 0.002012 0.001675 1.201 0.23292

AMD -0.077371 0.065114 -1.188 0.23797

DR -0.068182 0.053309 -1.279 0.20430

RVO -0.098845 0.073380 -1.347 0.18147

sex 0.019329 0.042595 0.454 0.65111

---

Signif. codes: 0 ‘***’ 0.001 ‘**’ 0.01 ‘*’ 0.05 ‘.’ 0.1 ‘ ’ 1

**Fatty acid: 18:3**

Coefficients:

Estimate Std. Error t value Pr(>|t|)

(Intercept) 0.509017 0.835487 0.609 0.5439

BMI -0.009837 0.025096 -0.392 0.6960

statin use 0.662007 0.281692 2.350 0.0210 *

fasting -0.018687 0.337388 -0.055 0.9560

age 0.001518 0.009274 0.164 0.8704

AMD -0.052647 0.360669 -0.146 0.8843

DR -0.056671 0.295281 -0.192 0.8482

RVO 0.822200 0.395615 2.078 0.0406 *

sex -0.374805 0.232479 -1.612 0.1105

---

Signif. codes: 0 ‘***’ 0.001 ‘**’ 0.01 ‘*’ 0.05 ‘.’ 0.1 ‘ ’ 1

**Fatty acid: 18:3 (omega)**

Coefficients:

Estimate Std. Error t value Pr(>|t|)

(Intercept) 0.194506 0.403436 0.482 0.6309

BMI 0.009211 0.012244 0.752 0.4539

statin use 0.009054 0.136049 0.067 0.9471

fasting -0.391044 0.163432 -2.393 0.0189 *

age 0.003140 0.004477 0.701 0.4849

AMD 0.372948 0.175422 2.126 0.0363 *

DR -0.082841 0.145535 -0.569 0.5707

RVO -0.148623 0.192646 -0.771 0.4425

sex 0.013124 0.113557 0.116 0.9083

---

Signif. codes: 0 ‘***’ 0.001 ‘**’ 0.01 ‘*’ 0.05 ‘.’ 0.1 ‘ ’ 1

**Fatty acid: 20:3**

Coefficients:

Estimate Std. Error t value Pr(>|t|)

(Intercept) 2.279100 0.669230 3.406 0.001 **

BMI 0.006023 0.020115 0.299 0.765

statin use -0.037374 0.231112 -0.162 0.872

fasting 0.428477 0.270115 1.586 0.116

age -0.006972 0.007427 -0.939 0.350

AMD -0.269432 0.288753 -0.933 0.353

DR -0.254008 0.236404 -1.074 0.286

RVO -0.388063 0.325412 -1.193 0.236

sex 0.198189 0.188893 1.049 0.297

---

Signif. codes: 0 ‘***’ 0.001 ‘**’ 0.01 ‘*’ 0.05 ‘.’ 0.1 ‘ ’ 1

**Fatty acid: 20:4**

Coefficients:

Estimate Std. Error t value Pr(>|t|)

(Intercept) 5.497286 1.140470 4.820 6.12e-06 ***

BMI -0.020453 0.034641 -0.590 0.5564

statin use 0.315457 0.394056 0.801 0.4256

fasting -0.949699 0.461749 -2.057 0.0427 *

age 0.007097 0.012653 0.561 0.5763

AMD -0.450167 0.495624 -0.908 0.3663

DR 0.468537 0.411189 1.139 0.2577

RVO -0.226872 0.559614 -0.405 0.6862

sex -0.179629 0.325833 -0.551 0.5829

---

Signif. codes: 0 ‘***’ 0.001 ‘**’ 0.01 ‘*’ 0.05 ‘.’ 0.1 ‘ ’ 1

**Fatty acid: 20:5**

Coefficients:

Estimate Std. Error t value Pr(>|t|)

(Intercept) 0.9043305 0.2842596 3.181 0.00204 **

BMI -0.0059886 0.0086343 -0.694 0.48982

statin use -0.0509181 0.0982177 -0.518 0.60550

fasting -0.0391771 0.1150898 -0.340 0.73438

age -0.0004546 0.0031536 -0.144 0.88573

AMD -0.0114229 0.1235332 -0.092 0.92654

DR -0.1059292 0.1024881 -1.034 0.30423

RVO -0.1120709 0.1394826 -0.803 0.42391

sex -0.0053462 0.0812131 -0.066 0.94767

---

Signif. codes: 0 ‘***’ 0.001 ‘**’ 0.01 ‘*’ 0.05 ‘.’ 0.1 ‘ ’ 1

**Fatty acid: 22:6**

Coefficients:

Estimate Std. Error t value Pr(>|t|)

(Intercept) 1.010148 0.300367 3.363 0.00117 **

BMI 0.004037 0.009224 0.438 0.66277

statin use -0.018100 0.105415 -0.172 0.86409

fasting 0.036492 0.120850 0.302 0.76344

age 0.002067 0.003335 0.620 0.53702

AMD 0.077707 0.130991 0.593 0.55464

DR -0.155405 0.111573 -1.393 0.16738

RVO -0.010508 0.147493 -0.071 0.94337

sex -0.089028 0.085901 -1.036 0.30302

---

Signif. codes: 0 ‘***’ 0.001 ‘**’ 0.01 ‘*’ 0.05 ‘.’ 0.1 ‘ ’ 1
